# Modelling the structure of object-independent human affordances of approaching to grasp for robotic hands

**DOI:** 10.1371/journal.pone.0208228

**Published:** 2018-12-26

**Authors:** Giuseppe Cotugno, Jelizaveta Konstantinova, Kaspar Althoefer, Thrishantha Nanayakkara

**Affiliations:** 1 Centre for Robotics Research, Department of Informatics, King’s College London, Strand, WC2R 2LS, London, United Kingdom; 2 Centre for Advanced Robotics @ Queen Mary (ARQ), Faculty of Science and Engineering, Queen Mary University of London, Mile End Road, E1 4NS, London, United Kingdom; 3 Dyson School of Design Engineering, Imperial College London, South Kensington Campus, London, United Kingdom; Szegedi Tudomanyegyetem, HUNGARY

## Abstract

Grasp affordances in robotics represent different ways to grasp an object involving a variety of factors from vision to hand control. A model of grasp affordances that is able to scale across different objects, features and domains is needed to provide robots with advanced manipulation skills. The existing frameworks, however, can be difficult to extend towards a more general and domain independent approach. This work is the first step towards a modular implementation of grasp affordances that can be separated into two stages: approach to grasp and grasp execution. In this study, human experiments of approaching to grasp are analysed, and object-independent patterns of motion are defined and modelled analytically from the data. Human subjects performed a specific action (hammering) using objects of different geometry, size and weight. Motion capture data relating the hand-object approach distance was used for the analysis. The results showed that approach to grasp can be structured in four distinct phases that are best represented by non-linear models, independent from the objects being handled. This suggests that approaching to grasp patterns are following an intentionally planned control strategy, rather than implementing a reactive execution.

## Introduction

Multifingered grasping in robotics is a widely studied problem. Despite this, a general solution has not been found yet. Over the course of time, different approaches were attempted to address the problem. Early focus [1] was on control algorithms for three or four fingered hands [2]. The approach was to optimise the placement of robotic fingertips on the surface of an object to achieve force or form closure with the hand grip. However, it is difficult to scale such approach to novel or a large number of objects, as it requires ad-hoc computations of the optimal placements. Recent attempts show that it is possible to optimise this [3]. Later approaches exploited a known characteristic of human grasping—grasping synergies [4]. It is the ability to move the fingers as a group rather than individually. This concept has been implemented in hardware [5, 6] and in software [7, 8]. Such approaches are simpler and easier to scale, but selecting the minimum number of synergies in a hand design [9] or formally assessing the quality of grasp [10] are still research topics in their early stage.

In order to perform robotic grasping of an object, it is first required to reach it with the end-effector. Controllers for reaching have better overall performance and are used in industrial set-ups. Direct reaching is mostly a solved problem [11], while constrained reaching, i.e. obstacle avoidance [12] or following a trajectory [13] or a strict time limit [14], is still considered a research topic. The combination of reaching and grasping controllers in robotics, instead, was not investigated as much. Reaching and grasping are often considered as separate control problems, although some studies from neuroscience of grasping suggest the contrary [15, 16]. The influence of reaching on grasping is often taken into account when designing [17] or controlling [8, 18] a robotic hand, but has not yet been quantified formally how the two motions influence each other. Therefore, it is worth to study the interactions between reaching and grasping. A good reaching might compensate for a bad grasping, or a different reaching trajectory might be required for a different grasp posture.

There are many studies that combine reaching and grasping to obtain a better understanding of the environment, to learn how to use an object or to guide the hand effectively. Although those studies do not openly discuss the interaction between reaching and grasping, they do take it into account. Often, perception and learning aspects are included in the analysis of the combination of reaching and grasping. Interactive perception is a technique that requires the robot to build a representation of an object by interacting with it and observing the outcome of its actions [19]. An application of grasping to use an object can be seen in [20] where authors are employing the technique to teach a robot how to use tools from perception and interaction. For instance, the technique can be used to determine interactively how to fold laundry [21] when combined with gaussian processes. The combination of manipulation and reaching in interactive perception is used to improve the knowledge of the environment, and to understand how to interact with it. Therefore, perception and learning are fundamental components of this technique. Another popular and similar technique is active vision. This methodology originally addresses complex computer vision problems by changing the view point of the camera [22]. Such technique can be used to optimise the number of processed frames needed to execute a grasp [23], or to generate grasping points on-line to guide visual servoing [24]. Hence, in this technique the interaction between vision and reaching is used to guide grasping. However, as the end-effector is mounted on the same arm, grasping is influenced by reaching as a result. The above approaches study the interaction between reaching and grasping but do not target the phenomena directly. In such way, it is difficult to understand the phenomena in depth and scale it for different objects and domains (areas of application). As such, the main disadvantage is that those approaches are tailored to the specific problem. Additionally, an intense use of learning, required by interactive perception, often needs long on-line training for parameter tuning or model definition.

To overcome those limitations, there is a growing interest in studying human affordances for object manipulation [25]. The term “affordance” has two different interpretations, one psychological and the other neuroscientific. The first interpretation [26], also named Gibsonian or object affordance, states that the affordance of an object is a list of potential uses that the object itself suggests or allows to the user. The neuroscientific interpretation, sometimes referred as grasp affordance, defines an affordance as a list of possible strategies to grasp an object. It is believed that a visual stimuli triggers a set of possible actions to be performed [27] and the specific motion implementation is selected in the primary motor cortex [28] as initiation of a voluntary action. Our definition of affordance is closer to the neuroscientific interpretation than to the psychological one. In this work, an affordance is defined as *a possible way to approach and to grasp an object in order to perform a predefined action*.

In our definition the action can be divided in two parts: approaching an object and grasping it. In this work, the term *approaching* is referred to the act of reaching an object with the intention of grasping it and using it to perform an action. This is different from the term *reaching* which implies an open-loop displacement of the hand to a defined position; for instance, it could be touching a surface, pressing a button or positioning an industrial robotic end-effector for soldering. It is important to stress this difference, since an open-loop reaching action or grasping an object without the intention of using it is not sufficient to obtain a grasp affordance. Indeed, an open-loop action as described above gives no guidance in selecting the most appropriate motion.

Affordances openly target the interaction between reaching and grasping to understand the phenomena of approaching to use an object. Grasp affordances focus more on the implementation facet of the action, while object affordances focus more on the cognitive and reasoning side. In one of the first studies [29] of implementation of object affordances, authors describe how optical flow can aid a robot to learn to roll an object from visual perception. A later study [30], uses Bayesian networks to infer the object affordance of specific objects from a restricted set of available actions that can be performed on them. In both cases the approach is tailored to the domain of use or to a limited set of features which is difficult to extend. Other studies proposed different approaches for representing a grasp affordance. In [31] authors encode a grasp affordance for a given object as a probabilistic gripper placement learned either from human imitation or an off-line model, and improved by experience to compensate for mismatches from the original model. In [32], an ontological approach is used to infer the most appropriate grasp affordance given a fixed set of perceived object properties. The common limitation of all the above studies is that the approaches are tightly coupling different aspects of affordances together, such as vision, learning and motion control. An affordance is composed by an interrelation of different features, such as perception, reaching and grasping. However, a strong coupling between features creates complicated and domain specific systems that are difficult to scale on a larger set of objects, properties or new domains [33, 34].

The focus of the above works is on specific manipulation tasks. Hence, it is difficult to design a general approach for a grasp controller that can manipulate novel everyday objects, which are designed for human use. In this respect, human studies can provide guidelines for future robotic implementations and several times this happened in the past [35]. For example, in [36], the authors are modelling human touch strategies of soft objects. The same model was later implemented on a robotic platform [37] with good results. Another example is shown in [38]. The authors perform human experiments of pick and place, grasping with sensory constraints, to identify the conditions that favour an action plan over another. The model that defines the conditions and the plans is general enough to be transferred to a robot with adequate sensing capabilities for grasping and reaching. This shows that human studies can be used to set a base for a robotic implementation or to guide the robotic learning.

This work establishes a first step towards a modular definition of grasp affordance, where different aspects, such as approaching and grasping, can be combined without the need of tailoring them to the specific domain of application. Our approach in this work is to analyse the approaching part of a grasp affordance from human demonstrations and to provide a model that describes the general pattern. This work is a fundamental study of human behaviour required for implementing robotic approaching to grasp controllers. For an initial robotic implementation, it is sufficient to provide a control strategy for displacing the end effector, in the form of a position or speed trajectory. At this stage, it is not required to take into account the end effector orientation as this can be derived in different ways by an existing controller, such as a Cartesian hand controller. In this paper, it is analysed the hand trajectory only of the motion, since the aim is to provide a base for object independent human-inspired grasp affordance controllers in robotics and further insights on the human motion.

This paper addresses the question of whether or not, a general, object independent pattern of the approaching part of a grasp affordance, defined previously, can be characterised and modelled from human demonstrations. As well as studies whether a grasp affordance approach motion is a planned strategy or it is a set of reactive adjustments performed during the execution.

The contributions of this paper are:

- The interaction between reaching and grasping is characterised by analysing human grasping experiments in terms of hand to object distance.
- The motion pattern structure is defined in terms of rate change of the distance and of the displacements of the fingers.
- A set of object-independent models is derived from the data to describe a general, object independent pattern of approach to grasp.

The proposed interpretation of grasp affordance can shift the attention from the specific object to grasp to how well the selected posture will perform the selected action, reducing the dependence on the specific domain of application.

The rest of the paper is organised as follows: in Section II the experimental data and data preprocessing methodology is discussed. Section III presents the results of the data analysis and describes the phases of approaching. In Section IV a set of models for the data is presented. Section V is the discussion and Section VI draws out the conclusions.

## 1 Methodology

### 1.1 Experimental protocol

The aim of this study is to understand whether humans have a general, object-independent pattern of approaching to grasp to perform a specific action and to model its structure. Additionally, it aims at providing fundamental insights on the structure of human approaching motion for future robotic control applications following a wider multidisciplinary approach. It is important to underline that a specific action to perform is needed, as this constraints the list of possible strategies and postures to the ones actually useful for the task. Not defining an action to be performed creates an open scenario where any approach to grasp strategy is acceptable. In this way, it is not possible to discriminate the most appropriate strategy and grasp affordance. The action selected for the task is hammering on a point. This action was selected because it is easy to generalise to similar actions, such as inserting. Additionally, hammering is one of the first actions ever learned by infants [39] and it was the action used by prehistoric humans for crafting the Oldowan stone tools [40], hence this action could also be used in other simple scenarios such as basic crafting.

Approach to grasp data and object motion data were collected from human trials for this study. For this purpose, it is important to track the hand, wrist and fingers motion in order to define trajectories and finger postures in a trial. Also the object position and orientation are tracked through the whole experiment. This is required in order to highlight the overall grasp affordance decision process by relating the object position and orientation to the grasping motion of the human participant, instead of processing the two independently.

A system of four motion tracking cameras (Vicon Bonita) was used for recording the object data at a capture frequency of 100 Hz. Additionally, a commercial arm and hand wearable fibre optic motion capture (MoCap) system (Measurand ShapeHandPlus) was used to record human data at a capture frequency of 77 Hz. Two sensing systems are required to ensure an accurate tracking throughout the experiment, both for the hand and the object. The use of a vision-based tracking system on its own [41] is subject to inaccuracies as the hand occludes the object when grasping. Those can be mitigated by forcing subjects to employ a limited set of grasping postures. This would influence the natural reaching to grasp motion and limit the analysis. On the other side, a data glove tracks the hand very accurately, but it provides little spatial information on the position of the object in the scene. As the analysis relies on the relationship between hand and object, those two entities have to be tracked and related accurately at all times to obtain meaningful observations. Hence both tracking systems have to be used together to allow subjects to perform natural motions while guaranteeing high accuracy, robustness of tracking and detailed data. The combined system is robust as data can be lost only if the object is occluded from the view of most cameras. As the cameras surrounded the subject, such events were infrequent. The number of frames lost by the MoCap system is negligible.

The capture frequencies of the two devices were aligned to 100 Hz through linear interpolation. The overall tracking error of the combined tracking systems was no more than 1.5mm.

**Fig 1:**
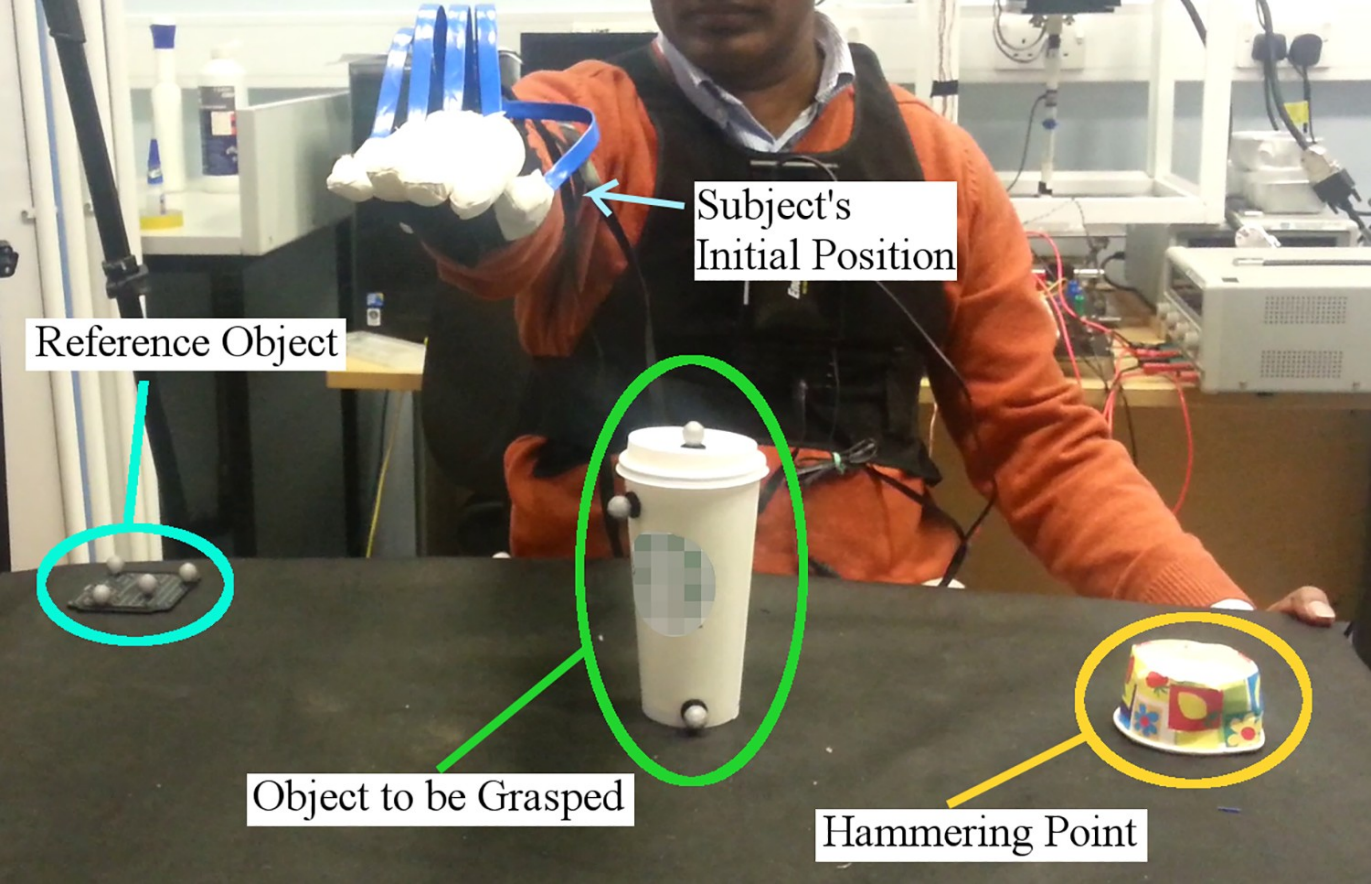
Illustration of the complete experimental set-up.

Participants were seated in front of a table and asked to wear the MoCap system on their dominant side. The table was placed in the centre of the field of view of the four cameras and it was covered with a black cloth to eliminate reflections from artificial light. The room was lit with artificial light only and the illumination was kept constant throughout a capture session. Fig 1 shows the complete set up.

**Fig 2:**
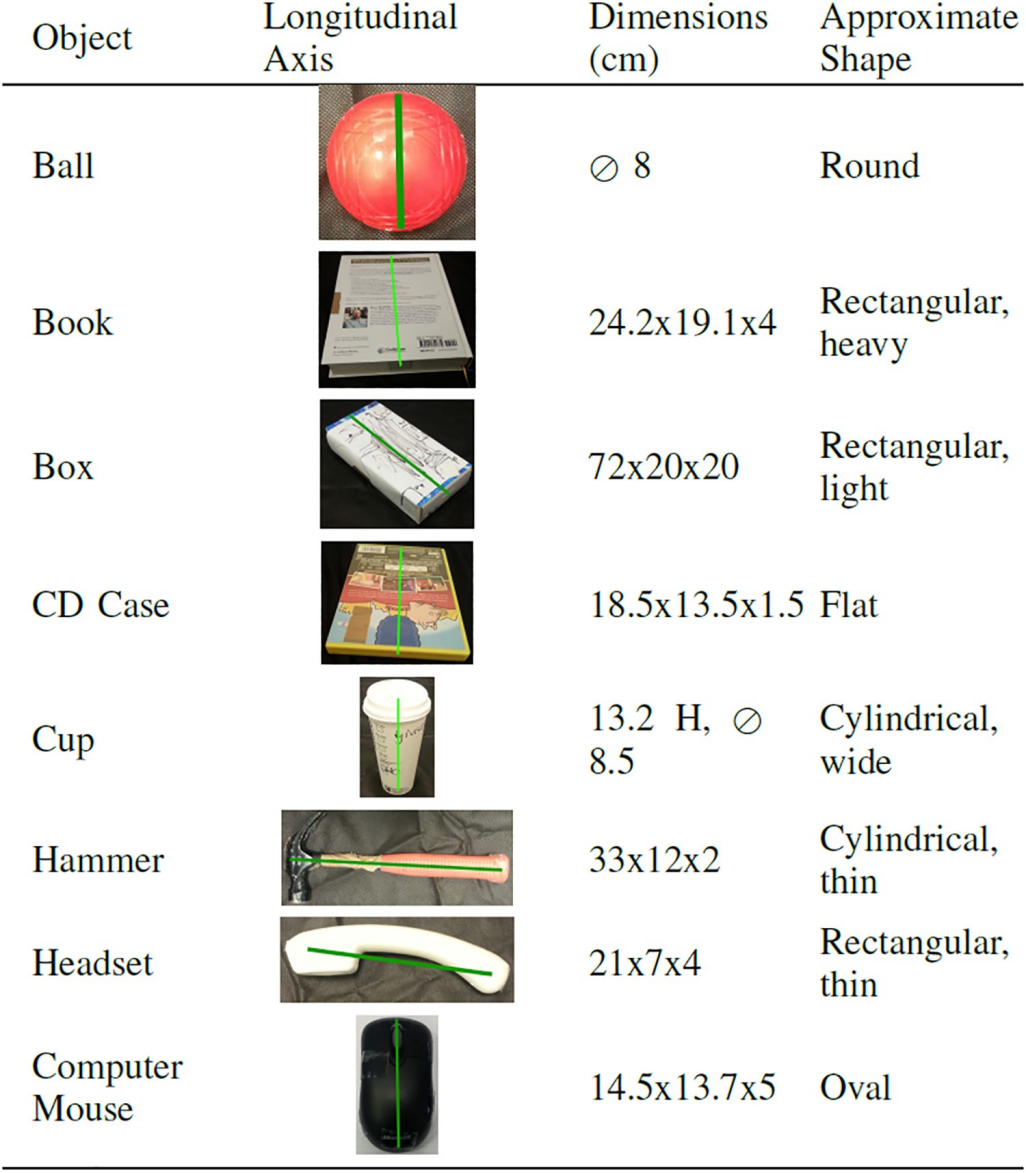
List of objects used in the experimentation and their properties. The longitudinal axis is highlighted in green on each object’s picture. The first dimension is along the longitudinal axis, the second is orthogonal to the axis and lying on the same plane. ⊘ stands for diameter, H stands for height.

A set of eight objects, with different shapes and weights, was used for the experiments: a plastic ball, a paper coffee cup, a card box, a phone headset, a CD keep case, an hard-cover book, a computer mouse and a hammer. The objects were selected to be of everyday use and to have different geometrical properties and weights. Fig 2 shows the properties of the objects. The aim was to make the action and the selection of the hammering surface non-trivial and to stimulate unusual approach to grasp strategies. In this way it is possible to define a general object-independent grasping pattern given the variability of approaches. The objects were given one by one to each subject in random order and, when applicable, random orientation—the longitudinal axis of the object was either parallel or orthogonal to the table edge.

Nine subjects, seven males and two females, performed the experiments. The study was approved by the King’s College London Ethical Committee, REC reference Number BDM/12/13-27, and participants provided written informed consent. The subjects were recruited as volunteers among the members of the Centre for Robotics Research in the Department of Informatics at King’s College London. The experiments were performed in the period from October to November 2015. Participants had no history of previous motor impairments and they were all right handed. The mean hand width was 79.7 mm, the mean hand length was 189.3 mm. The measurements were performed as in [42] based on hand breadth and length from digitizer. Each subject performed the experiments with the objects placed in two different orientations, when possible. The cup and the ball do not have a unique orientation due to their circular base. Each approach to grasp experiment was repeated two times. In total, 28 demonstrations were collected for each participant. The total number of trials collected exceeds those usually obtained in similar internationally recognised studies on human-inspired robotic control [7, 38, 43, 44].

For each participant, at the beginning, a hammering point on his non-dominant side was marked on the table with a paper cup as a damping place holder. The point was selected so that it would be easy to approach, grasp and hammer without the need of bending or rotating the torso. A small platform with 5 trackers was used as a common reference point for the Vicon and MoCap systems. It was placed on the corner of the table close to the dominant side of the subject. The position and orientation of the reference plate are fixed for the duration of the whole trial. The object to be grasped is positioned in front of the subject to allow comfortable approaching and grasping without the need of bending the torso. The participants are always able to perform a direct approach motion without avoiding any obstacle when performing the experiment.

**Fig 3:**
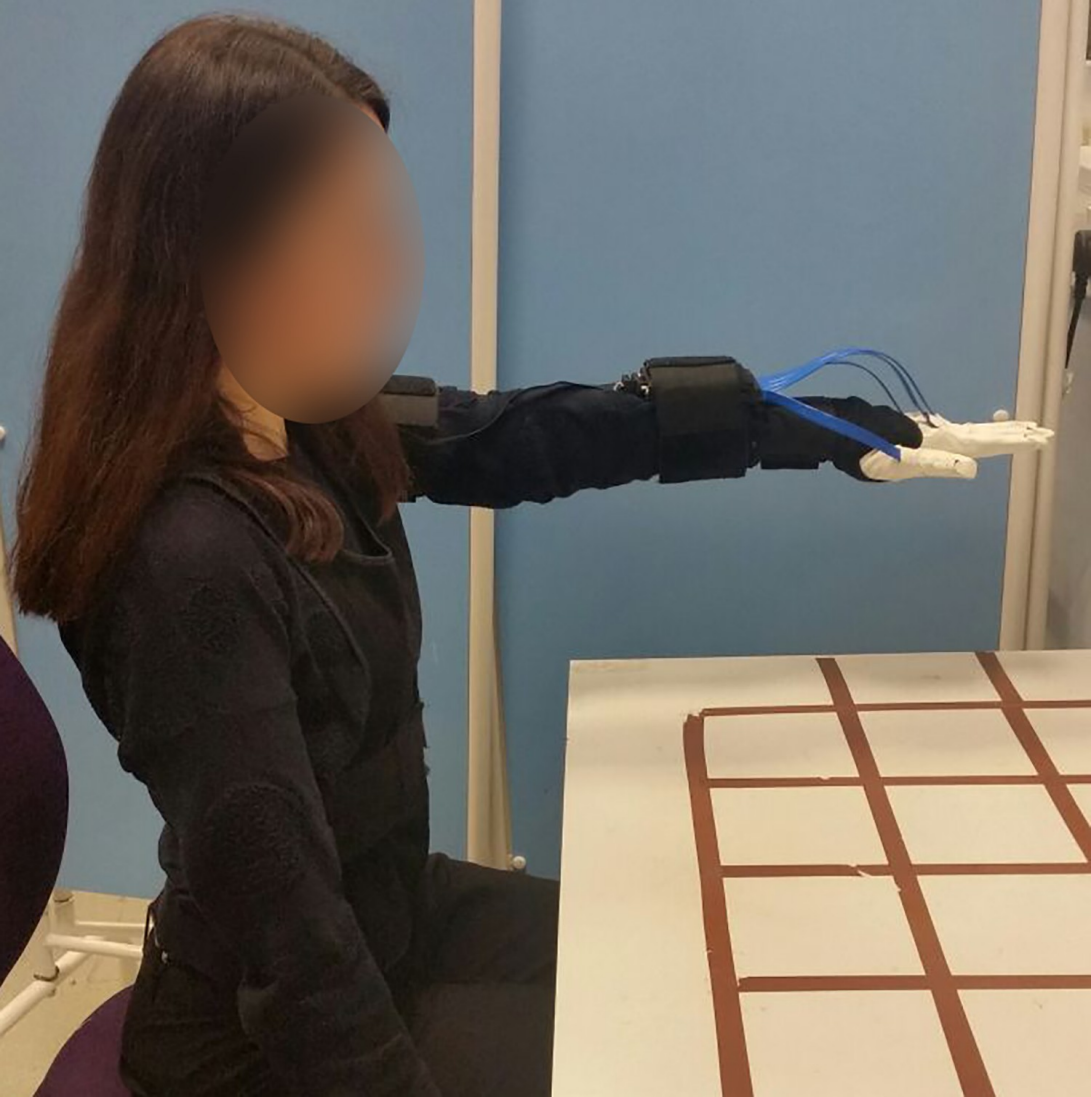
Initial reference posture: Arm in straight position parallel to the ground and orthogonal to the chest, hand fully open and flat fingers.

Subjects were shown a brief demonstration of the experimental protocol prior starting and they were asked if they had any question on how to perform the experiment. At the beginning of each trial, subjects adopted the initial reference posture shown in Fig 3. After adopting this initial posture, subjects performed the experimental protocol as follows:

1. Subjects covered the reference plate with their hand so that it is covered and not visually tracked. Losing the tracking allows to synchronise the starting point of the both data streams.
2. Subjects returned to the initial posture, so that the reference plate is tracked again (Fig 3).
3. Subjects approached the object naturally. No constraints or suggestions were given on which grasp affordance was the most suitable.
4. Subjects hammered the object on the area selected during the set-up. Subjects were free to choose the hammering style or point of contact with the hammering area.
5. Subjects placed the object away and the data collection was stopped.

If the visual tracking of the object was lost at any point during the experiment, the trial was repeated. The trial was repeated also if the tracking of the reference plate was lost at any time when not specified by the protocol.

Although the whole action listed above was captured, only the motion within step 3 was analysed in this paper. Performing the complete action was required to ensure that subjects would perform a natural approaching to grasp pattern, so that any difference with plain reaching could be highlighted.

### 1.2 Data processing

In the analysis the relationship between hand position and the object centroid is characterised.

The object and reference plate centroid positions were acquired directly from the visual tracking system with no need of further processing.

Instead, the positions of every joint of the kinematic model of the arm were collected during a trial. The hand position is defined as the centroid between the wrist, middle and ring fingers metacarpophalangeal (MCP) joint positions. The positions were derived from the kinematic model using the MoCap toolbox for MATLAB [45].

**Fig 4:**
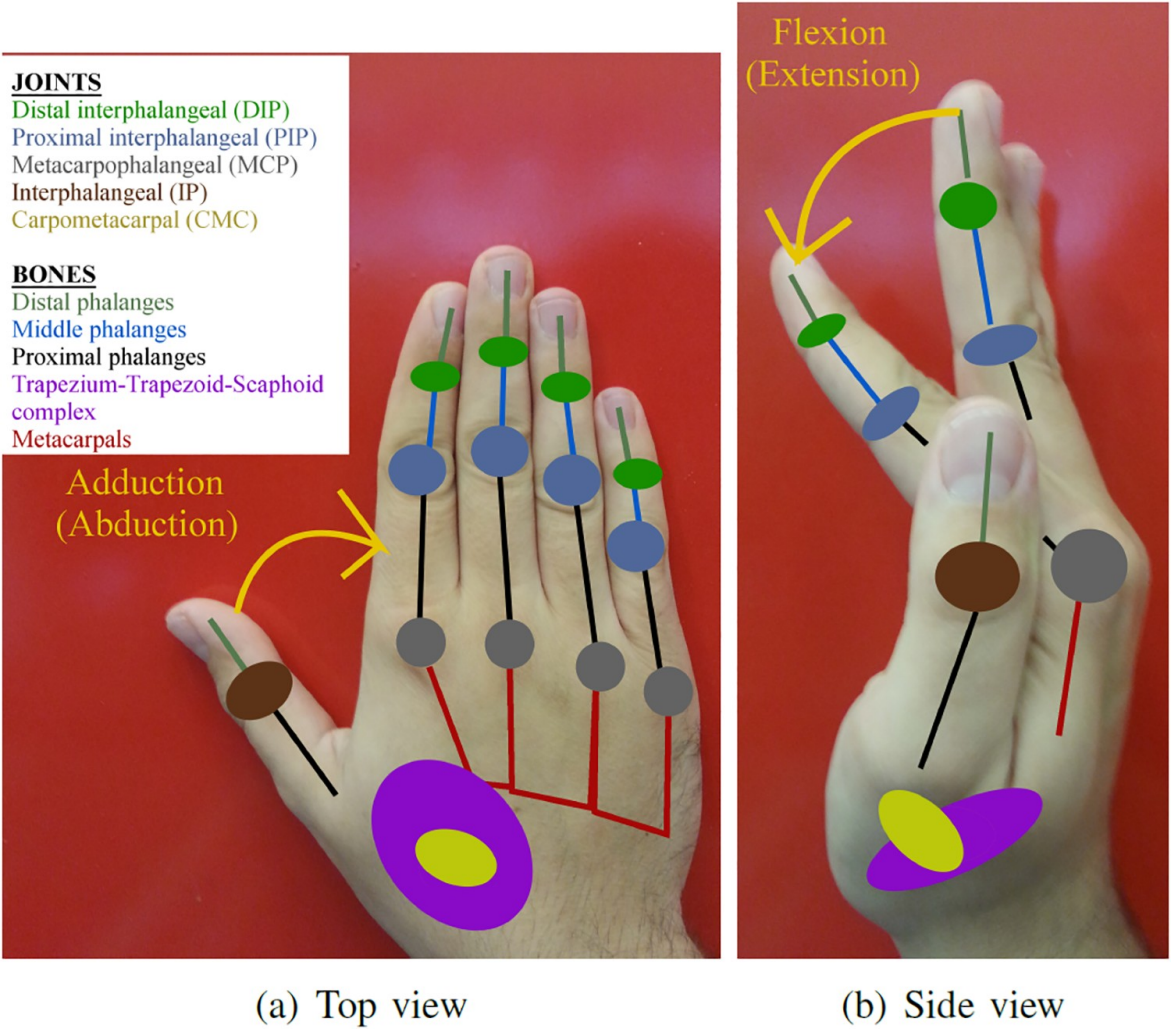
Schematics of the hand bones and joints involved in the capture.

Metacarpal, proximal and distal interphalangeal joint angles and metacarpal adduction/abduction joint angles of the index, middle, ring and little fingers were also collected (Fig 4(a) and 4(b)). As commercial MoCap gloves introduce inaccuracies when capturing thumb motions, the thumb joints were not collected. This choice do not influence the analysis, since the precise details of the hand posture are not considered. The fingers motion as a whole is examined to provide an additional qualitative description to the approach patterns. Only MCP flexion/extension joint motion of the fingers was analysed as it has the greatest impact on the motion of the whole finger [46]. The main focus of the analysis is the approaching distance of the hand to the object. As such, the motion of the fingers is to be used qualitatively as a reference and to provide further context to the reader during the analysis. Further in the text, the MCP flexion/extension data is referred as simply metacarpal data. The MCPs speed was obtained from the time derivative of the MCP position data and it was aggregated taking the mean as follows:

$$\begin{array}{c} f=\frac{\left(i_{t}^{\prime }+m_{t}^{\prime }+r_{t}^{\prime }+l_{t}^{\prime }\right)}{t_{tot}} \end{array}$$ (1)

Where $i_{t}^{\prime }$, $m_{t}^{\prime }$, $r_{t}^{\prime }$ and $l_{t}^{\prime }$ are the speed values at time *t* of the index, middle, ring and little fingers, while *t*_*tot*_ is the duration of the whole sequence. *f* is the mean MCP speed data shown in the analysis. The speed of each finger was derived from the original not normalised position data. Finger motion data was aggregated in this way to provide a clear and immediate qualitatively summary of the overall fingers motion during approaching.

The starting point of the data streams of the visual tracking and wearable MoCap were synchronised as part of the experimental protocol. The common starting point of the two capturing systems was the moment where the reference plate lost visual tracking as the subject’s hand covered its centre. Subjects must cover the reference plate centre to ensure that visual tracking is lost. As such the centroid of reference plate and the centre of the hand are always close to each other on the XY plane by design, reducing the reconstruction error. Additionally, a fixed offset, corresponding to the height of the reference plate’s markers, was removed from the wearable MoCap data. This allows to align the centre of the hand to the centroid of the reference plate also along the Z axis. The reference frame of the MoCap data was transformed to the reference plate coordinates in order to have a common base between the two capture devices and allow comparisons. The reference frame of the visual tracking system coincided with the reference plate centroid.

Although the full motion was captured, from the initial position to hammering; only the approaching part of it was analysed. Within a whole trial, the beginning of the approaching sequence was marked by the metacarpal and proximal joints of the fingers just starting to displace. The end of the sequence was marked by the object centroid being just displaced vertically (Z axis) as the hand started to lift the object. Table 1 summarises all the values used for segmenting the approach motion. The values in the table are normalised medians and variances. The rest of the motion was still required to make sure that subjects would perform a realistic approach to grasp as the object would effectively be used for hammering. The experimental protocol was designed to facilitate identifying and segmenting the approaching part of the motion. Also, using two different capturing systems allowed to discriminate different key moments univocally, as the same moment can be observed on both systems. The initial posture adopted by subjects is unusual when approaching to grasp since the arm and all the fingers are fully extended. Subjects immediately change their fingers configuration when initiating the motion. This moment marks the beginning of the analysed sequence and can be observed very clearly from the wearable MoCap system’s data. To further reduce the possibility of confusing a similar ambiguous posture with the initial posture, the protocol requires subjects to cover the reference plate prior adopting the initial posture. The only moment when the reference plate is not tracked is always at the beginning of the data collection. As such, it is straightforward to observe this moment in the visual tracking system’s data. Since the loss of the reference plate’s tracking happens seconds before adopting the initial posture, the beginning of the analysed sequence can be identified by combining the information obtained from both capture systems. Similarly, the end of the approaching motion can be identified by observing the data from the combined systems. During a trial, the object is still until grasped. Afterwards, the object’s centroid displaces vertically as the subject transports the object to hammer. The displacement of the centroid marks the end of the analysed sequence. At the same time, the subject’s hand is also displacing in the same direction of the centroid from a position close to the table’s surface. When both systems’ data show that hand and object are moving at the same time, it can be concluded unambiguously that the approaching motion is finished. Our approach is different from the state-of-the-art. Typically, other studies analyse the whole motion sequence which is designed to capture a single phenomenon [47–50]. The aim of this experiment is to highlight how all the factors in a complete action influence the approaching phase of the motion, rather than just studying approaching in isolation. Including other factors in the experiment is required as this study proposes an application to robotics. As such, considering the approaching motion individually would abstract important elements of the human behaviour, resulting in an approaching controller which might not work in real life.

**Table 1:** Summary of the median normalised values and variances of the quantities used for segmenting the approaching motion from a whole trial.

Quantity	Beginning	End
Index Metacarpal	−0.023 ± 0.04	−0.34 ± 0.41
Index Proximal	0.003 ± 0.006	0.91 ± 0.05
Middle Metacarpal	−0.003 ± 0.035	0.29 ± 0.48
Middle Proximal	0 ± 0.003	0.99 ± 0.02
Ring Metacarpal	−0.008 ± 0.047	0.90 ± 0.33
Ring Proximal	0.007 ± 0.006	0.99 ± 0.026
Little Metacarpal	0.07 ± 0.09	0.82 ± 0.37
Little Proximal	0.02 ± 0.02	0.99 ± 0.05
Object X Position	0.26 ± 0.01	0.27 ± 0.02
Object Y Position	0.66 ± 0.01	0.70 ± 0.03
Object Z Position	0.046 ± 0.002	0.13 ± 0.02
Palm X Position	0.79 ± 0.06	0.61 ± 0.07
Palm Y Position	0.39 ± 0.09	−0.40 ± 0.08
Palm Z Position	0.66 ± 0.06	0.27 ± 0.10

The normalised euclidean distance between centre of the palm and the object centroid was calculated and used to quantify the relationship between hand and object positions. The hand rotation and the hand-object angular relationships (azimuth and zenith) were collected but not analysed as they are out of the scope of this work. The normalised euclidean distance is defined as approach distance and it was calculated as follows:

$$\begin{array}{c} a=\sqrt{\sum_{t=1}^{n}{\left(\frac{O_{d}^{t}}{O_{d}^{mx}}-\frac{P_{d}^{t}}{P_{d}^{mx}}\right)}^{2}} \end{array}$$ (2)

Where *a* is the approach distance, $O_{d}^{t}$ and $P_{d}^{t}$ are the values of the object and palm respectively for dimension *d* observed at instant *t*. $O_{d}^{mx}$ and $P_{d}^{mx}$ are the maximum position values for dimension *d* of the object and palm. Finally dimension *d* refers to *x*, *y* and *z* axes.

The approach distance represents the distance between the centre of the hand and the centroid of the object. It is a quantity which changes over time, as the hand gets closer to the object. The approach distance is a relationship between the hand and the object and it is used to quantify how close the hand is to the object at a given time. The purpose of normalising only on the maximum value is to solely improve the presentation of the data by transforming it in a non-dimensional quantity. As all the collected data already lays within the same range, a full normalisation is not otherwise required for the analysis.

The normalised approach distance data was derived two times, to obtain speed and acceleration that were filtered with a moving average filter with span 7. The speed and acceleration were not normalised a second time once the time derivative of the original quantity was taken.

## Results

### 1.3 Statistical analysis

We conducted statistical analysis of behavioural data to test whether factors such as the grasped object, the preforming subject, or the specific execution influence the approach motion. As we are defining a common object-independent approaching to grasp pattern, those tests are required to verify whether every trial can be treated independently or whether all trials can be clustered and analysed together or in groups. As such, an Analysis of Variance (ANOVA) test was performed.

The statistical analysis was performed to understand whether specific features of approaching to grasp depend on the object, the specific trial or the performing subject. To this respect, the standard deviation of approach distance for each individual trial was normalised and used as dependent variable. The standard deviation describes the overall rate of change of the distance within a trial. The larger the standard deviation the more the distance was changing undergoing peaks and valleys. Therefore, it is useful to observe which factor influences the change of the approach distance. It is expected to see no significant difference across trials if the standard deviation is similar. The independent variables were the subjects, objects and trial number for an object-subject combination. The results of Shapiro-Wilk test demonstrated the normal distribution of the data. The three hypotheses were tested using a one way ANOVA with 1-degree-of-freedom test. A hypothesis is considered significant if the Fisher’s index (*F*) is bigger than *F* critical and the null hypothesis is rejected with 99.9% confidence level, which corresponds to a probability distribution (*p*) less than 0.001.

The variance of the approach distance did not show a significant dependence on the object being grasped (*F*_1,230_ = 1.75, *p* = 0.19) or the specific trial (*F*_1,230_ = 0.04, *p* = 0.85) but it did show a significant dependence on the performing subject (*F*_1,230_ = 4.93, *p* < 0.001). Therefore the performing subject is a determining variable in deciding the shape of the approach motion for grasping. This result can be explained as every person performed the experiment at his or her own pace. As some subjects were more careful or more confident, the speed of the execution changed although every object was grasped more or less in the same way.

Additionally, it was tested whether the grasped object influences the structure of the approaching motion for each subject. Every trial was segmented in four phases, which are described in Section 1.4 The data in the first three phases of the motion was tested individually for each subject, the fourth phase was not assessed since, as mentioned in Section 1.4, this phase is performed differently. Each segment of trial was normalised and interpolated to obtain a matching duration. Two tests were performed: it was tested the similarity across pairs of trial segments and the similarity between all trial segments and a global reference—the mean. If both tests score a value lower than 5% of the overall maximum motion, then the object does not influence the approaching motion of individual subjects. Firstly, the pairwise similarity was evaluated as the mean squared difference between every two segments of trial in the same phase from the same subject. If the grasped object does not influence the approaching motion, then the trials shall not be too different from each other. For each subject and phase, every trial section was combinatorially tested with the others for a total of 10044 unique tests. The pairwise difference was evaluated as follows:

$$\begin{array}{c} p=E[{\left(d_{1}-d_{2}\right)}^{2}] \end{array}$$ (3)

Where *d*_1_ and *d*_2_ are two segments of trial of the same subject, *E*[] is the mean value of the squared difference among the trials and *p* is the pairwise difference. A low pairwise difference implies high similarity between the two trials. The value of the difference is presented as a percentage of the total motion for clarity. The mean pairwise difference aggregated across all subjects and phases is 0.25% ± 0.54. The pairwise difference was within range [0.001 ± 0.001 0.004 ± 0.004] for phase one, [0.17 ± 0.21 0.98 ± 1.12] for phase two and [0.06 ± 0.06 0.66 ± 0.77] for phase three. Furthermore, for each performing subject, the variance of the segments of trial within each phase was calculated, and it is also reported as a percentage over the total motion. If the grasped object influences the approaching motion, it is expected to observe a high variance as the trials overall significantly differ from their mean. The variance for all the subjects is in the range [0.05 0.20] for phase one, [0.14 0.80] for phase two and [0.03 0.36] for phase three. Since both tests demonstrated that the trials are at most 2% different from each other and no more than 1% different from the mean, if grouped by subjects, we can conclude that the grasped object does not influence the motion.

These results shows that the data can be grouped by subject for the analysis as the performing subject is a factor that influences the characteristics of the motion. These findings provide fundamental insights for robotics as they allow to derive approaching models which are generalisable across different objects. In Section 1.4, for the purpose of characterising the data, each trial has been analysed individually to provide a more detailed and granular analysis, as well as to avoid possible approximations. Discussions on the common features seen in the subjects’ data are drawn by observing all the trials individually and relating the single trials to each other by subject.

### 1.4 Characterisation of approach patterns

In this section the analysis of the data is discussed and a common structure of the approach motion is formulated. The data is analysed by observing the individual patterns of motion of the approach distance speed, acceleration and fingers speed variability. As the purpose of this study is to analyse approach motions of the hand to the object, the speed of the approach distance is the fundamental quantity analysed since it represents the rate of change of the distance between palm and object over time. The acceleration is considered to highlight changes in this fundamental quantity and to give structure to the motion. The hand position during approaching is considered, but not reported as it does not highlight well enough the dynamic changes that are involved during approaching. For similar reasons, the speeds of the metacarpophalangeal (MCP) joint displacement of the index, middle, ring and little fingers are analysed. To better highlight the moments where the MCP joints displace the most, the variance of the mean of the four fingers across the motion is examined. A high variance indicates a part of interest for the analysis since large MCP joint displacements imply that the hand is performing an activity such as preshaping.

**Fig 5:**
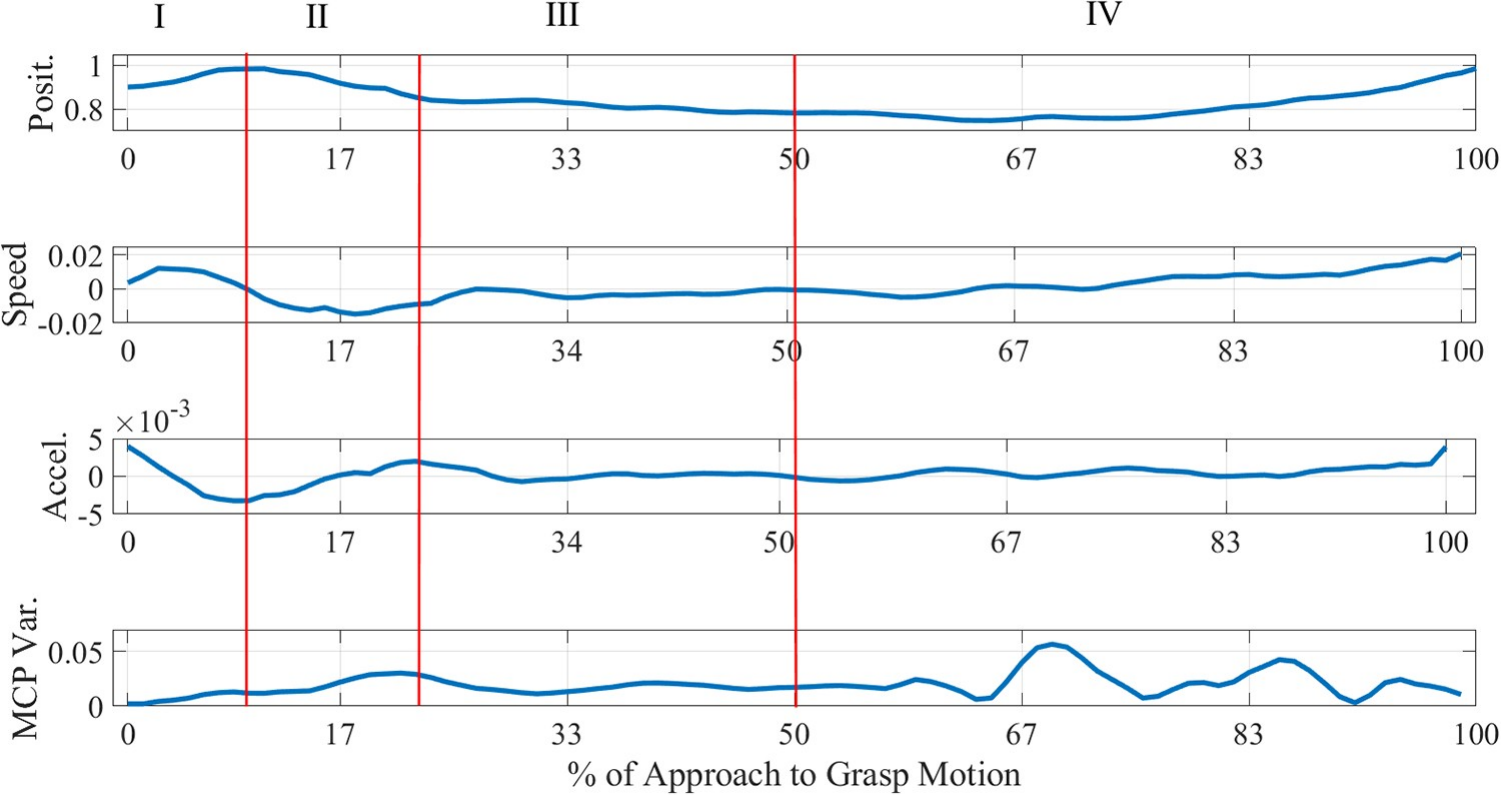
Sample approach to grasp trial. From top to bottom plot: hand position, speed, acceleration and variance of the four metacarpophalangeal joint speeds for the whole approach to grasp motion. The first data point corresponds to the moment the hand and the finger start to move, the last 17% of the motion shows the object being lifted for hammering. The Roman numbers identify the four phases.

Empirical comparisons of each trial showed that we can discriminate four phases. The first three phases represent the approach to grasp motion. The last phase comprise the final stage of grasping, when the object is firmly enveloped by the fingers, and the beginning of the lifting motion of the object is performed prior hammering. Each phase, except for the last, has its own characteristics which are common across all the trials. Fig 5 shows a sample trial from the dataset.

To demonstrate that the data is similar across the dataset, a correlation analysis is performed. The analysis is performed on positional data since it is the least processed data. The main issue to address is that subjects were performing the experiment at their own pace, hence the length of a phase or of the whole experiment is influenced by external factors such as the subject’s emotions (rush, boredom, etc.). For this reason, the four phases are analysed independently, and the duration of each phase for different trials are matched through interpolation. In this way the pattern structure within the phase is preserved. Each trial is segmented one by one according to the criteria defining each phase. The pairwise correlation coefficients for all the trials are calculated and the overall median value of all the coefficients is taken. The correlation coefficients for the first three phases are 0.93 (*p* < 0.000001), 0.99 (*p* < 0.000001) and 0.97 (*p* < 0.000001) respectively. This shows that the observed structure of the motion and the characteristics of each phase are common across all trials irrespective of the subject and the approached object. The fourth phase, instead, shows a median correlation coefficient of 0.16 (*p* < 0.00001) demonstrating that this phase is performed in different ways. This result is interesting since this phase features a high MCP joint speed variability which suggests that the fingers have a significant role in finalising the grasping, but the low correlation coefficient indicates that the hand position plays an important role as well. Since the focus of this work is on the first part of the approach to grasp motion, this feature is not discussed further and will be analysed in future work.

**Fig 6:**
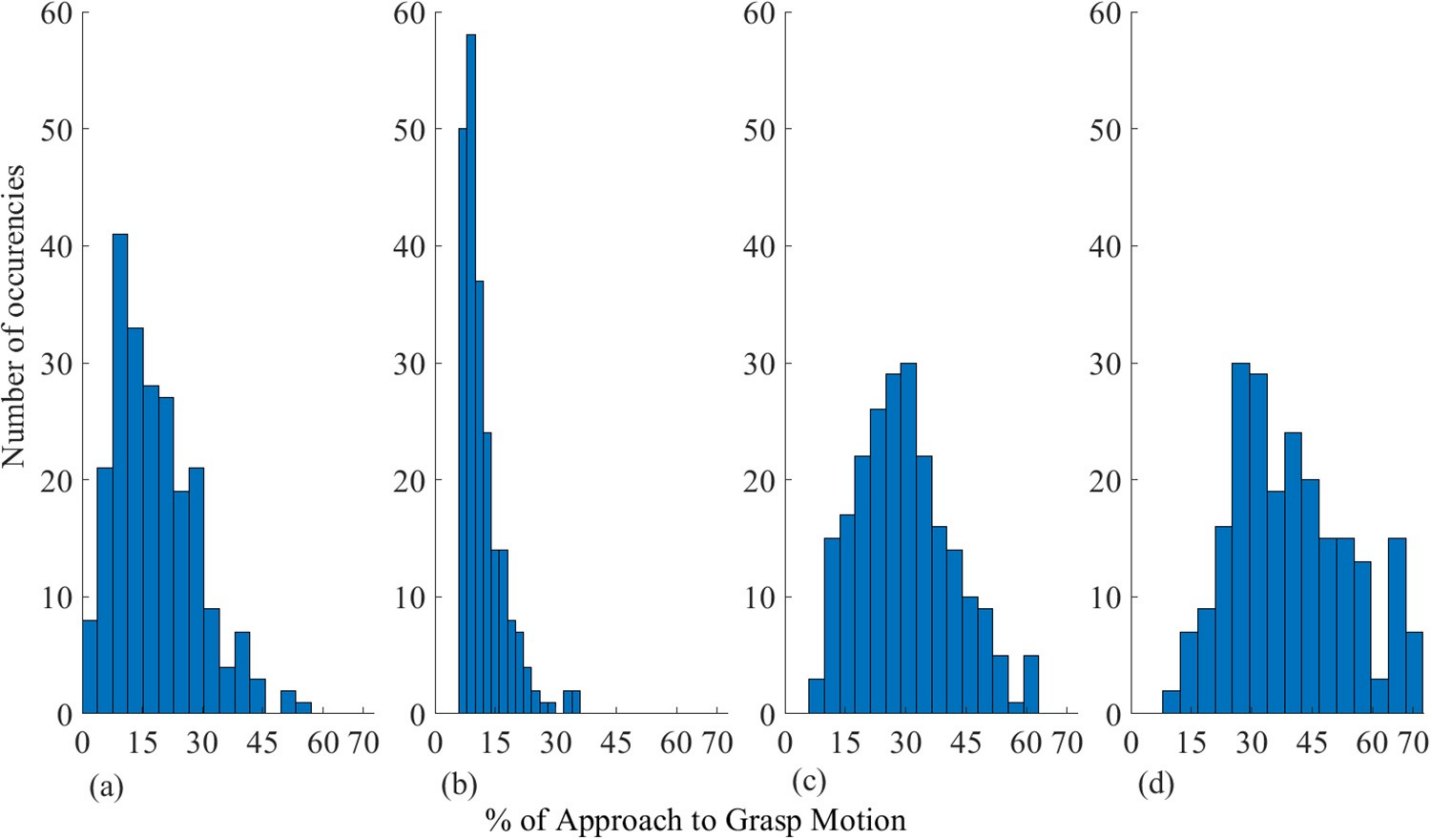
Distributions of phase coverages with respect to the whole motion. The abscissa (X axis) indicates the percentage of coverage of the whole motion, while the ordinate (Y axis) describes the number of trials with that coverage. Graphs (a), (b), (c), (d) show the distribution for phase one, two, three, four.

Below, the four phases are discussed more in detail. The features used to distinguish the four phases within the data are summarised at the beginning of each section. The value of the acceleration was used to split the data in phases. The average coverage of a phase is calculated from the corresponding lengths of every individual trial. The distribution of the lengths of each phase is summarised comprehensively in Fig 6. Since the data can be aggregated per subject (Section 1.3), common features within subjects are presented as such when appropriate, although each trial was observed individually.

#### 1.4.1 First phase

In the first phase, the hand starts its approach motion to the object and the finger MCP joints just begun to displace. This phase covers as average 17.86% ± 6.92% of the total motion across subjects. The distinctive features of this phase are as follows:

- the hand speed increases to a peak and then starts decreasing;
- the hand decelerates abruptly until its global minimum;
- finger posture starts to shape from the initial flat hand configuration.

The value of the speed peak is independent from the object approached. Although the MCP joints are moving, their variance is not notably larger as the maximum mean peak in this phase is 22% times smaller than the global mean maximum. This suggests that the fingers are displacing because the preshaping is just started. Most of the preshaping motion is performed in the next phase. Another notable characteristic is that the speed profile is a bell-shaped curve resembling a Gaussian. This profile is distinctive for open loop reaching motions, as found in [51]. The main difference is that, in this case, the bell-shaped profile terminates before the whole motion is completed, while in [51] the profile is extended until the end of the motion. This suggests that subjects treat differently an open loop reaching motion from a targeted approach motion.

The moment when the acceleration reaches its global minimum indicates the end of the First Phase and the beginning of the Second.

#### 1.4.2 Second phase

In the second phase, most of the preshaping is performed and the hand motion patterns undergo important changes in speed and acceleration. This phase covers as average 12.03% ± 2.10% of the total motion across subjects. These features characterise this phase:

- the hand speed stays within its global minimum range;
- the hand acceleration increases until its first peak;
- most of the preshaping is performed, as fingers’ MCP speed variability is increasing.

In most trials in this part of the motion, the MCP speed joint variance is above 72% of the total variability, suggesting that most of the preshaping is performed in this phase. Indeed, the only other moment when the MCP variance is higher is in the fourth phase. This indicates that subjects select the finger posture to be used for grasping by the end of this phase.

Additionally, for each individual subject, the variability of his speed patterns undergoes a bell-shaped increase, underlining that the hand approach pattern is also adjusted in this phase. Therefore, different people perform this phase in different ways. Additionally, in this phase, the subject adopts the actual hand approach pattern and the finger posture to use for grasping. This suggests that the approach to grasp motion is decided and adjusted on the way rather than being preplanned. In next section it is discussed whether the adjustment is reactive or intentional.

The moment when the acceleration reaches its first peak corresponds to the end of the Second Phase.

#### 1.4.3 Third phase

In the third phase, the distance between the hand and the object reduces until the approaching motion is terminated. This phase covers as average 30.05% ± 3.75% of the total motion across subjects. The features of this phase are the following:

- the hand steadily increases its speed until settling down to 0 (±0.001) mm/sec;
- the acceleration slowly converges to a steady state value of 0 (±0.0001) mm/sec;
- the hand closes up the distance with the object to finalise the grasping, as the fingers’ MCP joints speed variability change is minimal.

In this phase the finger MCP joint speed variance also greatly reduces until reaching a steady or null speed in some cases. This indicates that the implementation of the finger posture, selected in the previous phase, approaches its end until the fingers stop moving. This happens just before the actual grasp, where the fingers are enveloping the object, is performed.

The hand approach speed and acceleration also settle down to a more predefined pattern since the variability of those two quantities greatly reduces. It can also be observed that the speed pattern converges exponentially to a steady state value. Such change is observed in all the trials, although the time required to reach the settling value might change. This confirms that in this phase the approach pattern and finger posture strategies are implemented since, by the end of this phase the hand is steady and the fingers are not displacing, as they are ready to clamp the object.

The time when the acceleration and the speed settle down to the near zero steady state is the end of the Third Phase and the beginning of the Fourth Phase.

#### 1.4.4 Fourth phase

In the fourth phase the approaching motion is completed and the object is constrained in the hand and can be lifted for the subsequent action—hammering. This phase covers as average 40.06% ± 7.18% of the total motion across subjects. The common characteristic is that fingers’ MCP speed variance is changing as the final enveloping and in-hand adjustments of the object is performed. Also the hand speed might show a sharp increase in the final part as the object is lifted. Such increase marks the end of the Fourth Phase. This phase is the only part of the motion that is different across trials, and is not possible to establish any common feature in the hand motion patterns as in some trials the speed was steady in others the speed had oscillatory components.

This phase coincides with the second part of our definition of grasp affordance, where a specific grasp posture is employed on a precise part of the object. As such, the characterisation of this phase is beyond the scope of this work.

## 2 Modelling of approach to grasp

### 2.1 Methodology

Different model types were fitted to the speed of approach distance. The reason for using the speed of approach for modelling is that this paper aims at reliably describing the pattern of motion from human data and at providing a model as first hypothesis for an object-independent robot controller. Since a robotic end effector can be velocity controlled, a model based on the speed of approach can be easily implemented on a robotic counterpart.

The approach distance data was divided in the four phases mentioned in section 1.4 and models up to the fourth order were fitted. As the approach to grasp part of the grasp affordance is modelled, the fourth phase was not considered in the analysis.

224 trials were fitted one by one to estimate the approach distance models, while 28 trials were discarded as not suitable for model fitting due to missing data or noise. The trials discarded are randomly distributed in the entire dataset and do not relate to a specific subject or object. 75% of the dataset was used as training set and the other 25% was used as a test set. To reduce the bias from the specific data collected, 10 different test sets were randomly selected. The 10 test sets were used to perform cross-validation and to evaluate the quality of the fit. The length of the trials was normalised for each phase. In total, for each combination of model type and order, 1680 fits were performed including all the test sets. The set of parameters of a model undergoing cross-validation are the medians of the parameters resulting from the individual fits on the training set.

To evaluate the quality of fit, the R-squared value of the training set and root mean squared error (RMSE) of the test sets were evaluated. Additionally, a measure of model instability was defined. A model-order combination is considered unstable if the mean RMSE is fluctuating across the 10 test sets. In other words:

$$\begin{array}{c} U=\sum_{i=0}^{9}|E{\left[RMSE\right]}_{i}-E{\left[RMSE\right]}_{i+1}| \end{array}$$ (4)

Where *E*[*RMSE*]_*i*_ and *E*[*RMSE*]_*i*+1_ are the overall mean RMSEs resulting from the i-th and (i+1)-th test sets fitted to a given combination of model type and order, and *U* is the instability index: the larger the less consistent is the model-order combination. The measure of model instability is used to discard those models whose performance was inconsistent due to randomness of the heuristic calculation of the parameters. As the instability index is a measure of consistency, it is required to evaluate the impact of randomly selected test sets on the fit quality. The assumption is that if a model truly describes the natural phenomena it is less likely that it will perform very differently on different combinations of the test set. The index privileges models which show a similar RMSE score on all tests sets. To further guide the selection, the mean of the RMSE across all the 10 test sets was also evaluated. This is a summary of the model combination overall performance which can be used to identify the worse performers, which are the model combinations with a lower overall mean RMSE. The overall variability of the same value is also used to guide the selection if models have similar scores. This criteria is also used to loosely enforce consistency over different test sets.

A combination of model type and order was selected based on the following criteria:

- The R-squared of all available model combinations is compared and all models which scored less than 0.7 are discarded due to poor fitting.
- The overall mean RMSE is compared across the remaining combinations, those with RMSE larger than 0.0075 are discarded.
- The instability of each remaining combination is compared, models that score greater than 0.0005 are discarded.
- If a clear winner does not stand out yet, worse performers are discarded.
- Variance of the overall RMSE is assessed to provide hints to guide the selection at this point.
- If two models score equally the combination with least parameters is selected.
- A simple model is also selected earlier in the process if other models with similar scores have much more parameters.

**Fig 7:**
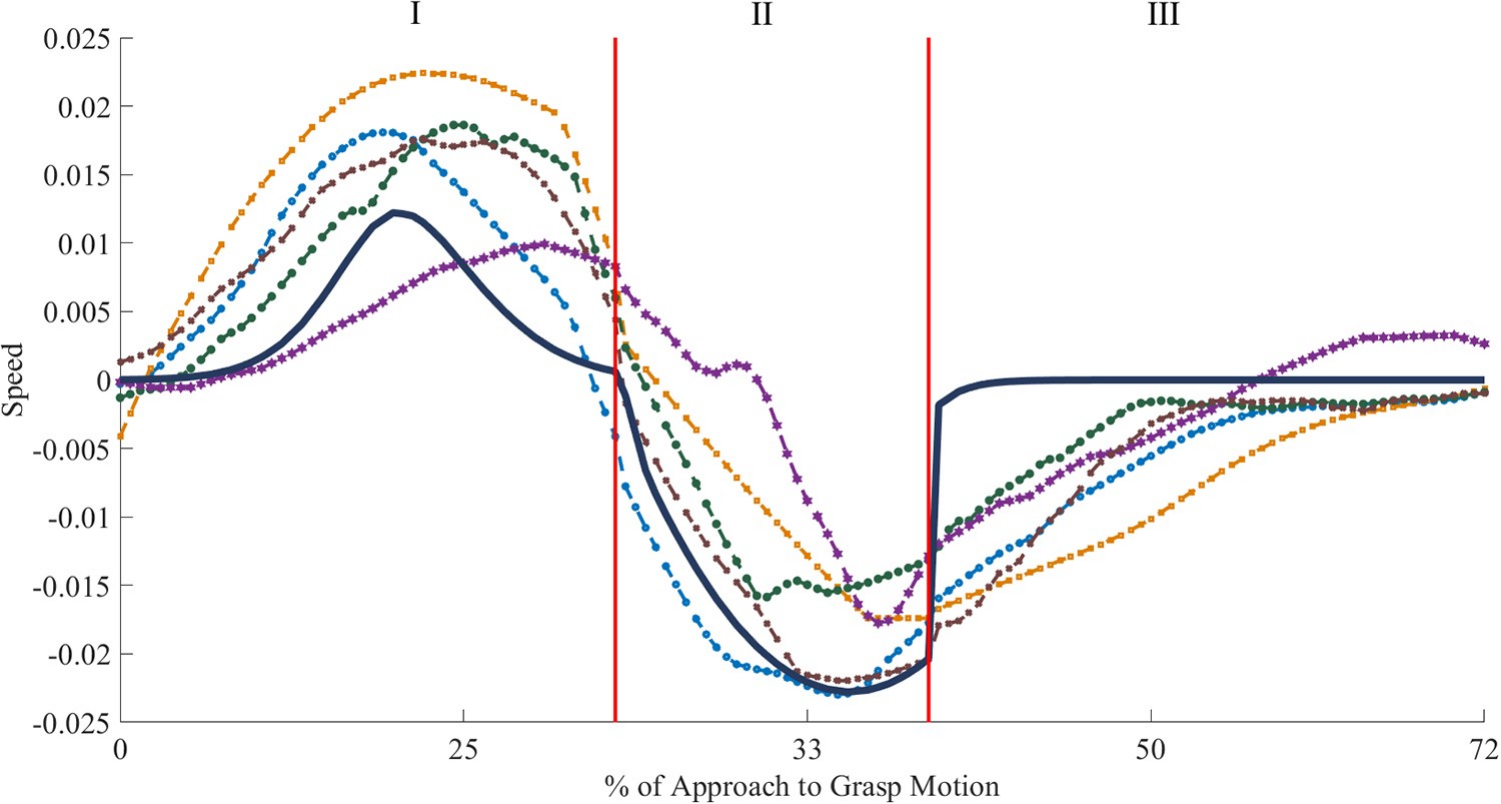
Combined output of the model fitted to each phase: Gaussian, polynomial and exponential. The model output (thick dark blue line) is overlaid on sample interpolated trials, used for fitting. Each dashed plot represents a different subject approaching different objects.

The thresholds and criteria chosen aimed at reducing the risk of selecting a model which would overfit the data or which would perform very well because of a lucky combination of trials used for training or testing. As such multiple factors are evaluated and the selection often is a trade-off, since rarely there is a single model that is the overall best. The most important criterion is the R-squared score as a low R-squared indicates a poor fitting. This threshold was selected according to state of the art machine learning practices. The thresholds for the RMSE and stability values were selected experimentally to discard the models with very poor fitting performance. The R-squared threshold was selected based on the variance of the data, in order to be strict enough to discard bad fits but not too strict prevent over-fitting. Overall, it was followed the Occam’s razor criterion [52], which privileges models with less parameters if the performances are similar. Within a selected model combination, the actual instance adopted as model for the first phase is the best RMSE fit across the 10 test sets. The model types used in the fitting were pre-selected by observing the shape of the median trial for each phase. A model type was selected either because the data was similar to its canonical output or because other studies used that model. For example, if the data did not show oscillatory components, the preliminary fit of a sinusoidal model to the median data would already be too poor to justify a thorough examination. Moreover, an oscillatory model would create jerky motions and unstable control, making it unsuitable for a real-world implementation. The classes of model type selected for fitting were Gaussian Mixtures (abbreviated to n-th order Gaussian) and Polynomials, for all phases, and, additionally, Exponential models for the third phase. All the models were intentionally selected in order to be expressed in closed form. The reason is that a relatively simple model can be automatically optimised at compile-time, when coded in software, or can be implemented easily in a reconfigurable hardware circuit (i.e. Field Programmable Gate Array), providing high performances for limited costs [53]. For each phase, the most reliable model is indicated in the table in bold, and a second best model is reported in italics, if present. The latter is the best model if the requirement on the instability index is relaxed. This can be interpreted an approximation of the original behaviour since it has generally a simpler structure than the most reliable model counterpart. As such, the models which comply to all the criteria specified are called most reliable models and are the models discussed in the paper. The second category of models, which ignore the instability requirement, are named approximated models and information on the this category is provided as a reference and is discussed in this section only. The optimal coefficients for each selected model combination are shown in Table 2, while the complete fit is shown in Fig 7.

**Table 2:** Optimal coefficients of the models selected for each phase for the most reliable and approximated models.

Coefficients	Phases		
	First	Second	Third
	**Most Reliable Models**		
*a*_1_	1.62 10^−3^	0.41 10^−4^	−1.73 10^−4^
*b*_1_	26.18	N/A	-0.7063
*c*_1_	4.95	N/A	N/A
*a*_2_	5.77 10^−3^	−18.08 10^−4^	−30.89 10^−4^
*b*_2_	26.97	N/A	-0.4122
*c*_2_	8.396	N/A	N/A
*a*_3_	5.246 10^−3^	−30.16 10^−4^	N/A
*b*_3_	29.65	N/A	N/A
*c*_3_	12.05	N/A	N/A
	**Approximated Models**		
*a*_1_	−0.15 10^−4^	0.41 10^−4^	−5.18 10^−6^
*a*_2_	5.93 10^−4^	−18.08 10^−4^	5.59 10^−4^
*a*_3_	−3.92 10^−4^	−30.16 10^−4^	16.98 10^−4^

### 2.2 Model validation

#### 2.2.1 First phase

The modelling of the first phase studied whether the approach velocity patterns were more similar to a Gaussian, as described in [51], or to a polynomial and which complexity for each model type is required to represent most reliably the used data. In this respect, many variants of Gaussian and polynomial models were discarded. The full details are shown in Table 3. The polynomial models were discarded from the model combinations that fulfilled the criteria set in section 2.1. Those models were mostly too unstable and inconsistently performing across different test sets or they performed poorly. This confirmed that the most reliable representation of this pattern follows a Gaussian. The best trade-off between complexity and reliability was a 3rd order Gaussian model. This model has nine parameters and a stability index of 0.38, while the alternative candidate, 4th order Gaussian model, has 12 parameters and a stability index of 0.25. Following the Occam’s razor criterion, the best trade-off is the 3rd order Gaussian model, since it saves 3 parameters at the cost of 0.13 stability, 0.04 points per parameter. The result of the fit can be observed in Fig 7.

**Table 3:** Summary of model fitting results of first phase data. Model type-order combinations with R-Squared less than 0.7 were omitted due to poor fitting. The selected most reliable combination for the phase is highlighted in bold, the approximated combination is highlighted in italics.

Type	Order	# Pars	*R*^2^	E[RMSE] (10^−3^)	Var (10^−5^)	Instab. (10^−3^)
Gaussian	2	6	0.78	7.07	1.50	0.59
	**3**	**9**	**0.79**	**7.22**	**1.46**	**0.38**
	4	12	0.81	7.14	1.59	0.25
Polynomial	*2*	*3*	*0.84*	*6.90*	*1.89*	*0.92*
	3	4	0.93	8.75	2.50	4.67
	4	5	0.97	7.27	2.00	4.96

The selected variant of the 3rd order Gaussian model is shown below, while the optimal coefficients are shown in Table 2.

$$\begin{array}{c} f\left(t\right)=a_{1}\;e^{-{\left(\frac{t-b_{1}}{c_{1}}\right)}^{2}}\;+a_{2}\;e^{-{\left(\frac{t-b_{2}}{c_{2}}\right)}^{2}}\;+a_{3}\;e^{-{\left(\frac{t-b_{3}}{c_{3}}\right)}^{2}}\; \end{array}$$ (5)

#### 2.2.2 Second phase

The model fitting of the second phase studied whether the human patterns were more similar to a Gaussian or a polynomial model and which complexity can appropriately describe the data. The models admitted to the selection showed all a good RMSE performance decreasing for some more complex variants of the models, as shown in Table 4. The Gaussian models were all discarded due to instability and inconsistency across different test sets, poor performance or too high complexity compared to the polynomial model with similar performance. Within the polynomial models, the 2nd order polynomial has shown to be the most reliable and simple version of polynomial models but still showing a good R-squared performance on the training sets. Therefore this part of the motion can be approximated with a polynomial:

$$\begin{array}{c} f\left(t\right)=a_{1}\;t^{2}\;+\;a_{2}\;t\;+\;a_{3} \end{array}$$ (6)

**Table 4:** Summary of model fitting results of second phase data. Model type-order combinations with R-Squared less than 0.7 were omitted. The selected combination for the phase is highlighted in bold and is equivalent for most reliable and approximated models.

Type	Order	# Pars	*R*^2^	E[RMSE] (10^−3^)	Var (10^−5^)	Instab. (10^−3^)
Gaussian	2	6	0.77	7.31	3.04	0.31
	3	9	0.84	7.28	3.18	0.33
	4	12	0.91	7.67	3.54	1.32
Polynomial	**2**	**3**	**0.95**	**7.25**	**3.12**	**0.06**
	3	4	0.98	7.55	2.92	1.23
	4	5	0.99	7.35	3.02	1.09

The optimal coefficients of this model are shown in Table 2 and the result of the fit can be observed in Fig 7.

#### 2.2.3 Third phase

The nature of the motion in this phase requires a rapid convergence to near zero speed, since the hand is quickly approaching the object to finalise the grasp mostly using the fingers. For this reason, exponential models were also fitted. This can be used as an assessment of how likely subjects were targeting the object with a quick reactive motion stopping the hand on contact. The results of the fitting, shown in Table 5, demonstrate that all the models admitted to the selection performed well in terms of RMSE on the test set, therefore this measure was not a discriminant. The Gaussian and the polynomial models were both discarded since they all obtained a too low stability score despite their RMSE values being within range. Therefore the pattern is represented by an exponential model of second order, since the first order variant obtained an R-squared score on the edge of the minimal criteria for admission. It can be concluded that subjects do approach the object with a quick and direct reactive motion rather than with a planned motion as for the other phases.

**Table 5:** Summary of model fitting results of third phase data. Model type-order combinations with R-Squared less than 0.7 were omitted. The selected most reliable combination for the phase is highlighted in bold, the approximated combination is highlighted in italics.

Type	Order	# Pars	*R*^2^	E[RMSE] (10^−3^)	Var (10^−5^)	Instab. (10^−3^)
Gaussian	3	9	0.69	4.49	1.28	0.46
	4	12	0.71	4.46	1.14	0.55
Polynomial	1	2	0.69	4.41	1.19	0.28
	*2*	*3*	*0.85*	*4.29*	*1.23*	*0.65*
	3	4	0.93	4.70	1.04	4.41
	4	5	0.97	5.01	1.21	6.56
Exponential	1	2	0.69	7.40	1.03	0.04
	**2**	**3**	**0.81**	**7.41**	**1.03**	**0.13**

The final Exponential model is shown below, while the optimal coefficients are shown in Table 2 and the result of the fit can be observed in Fig 7.

$$\begin{array}{c} f\left(t\right)=a_{1}\;e^{b_{1}\;t}\;+\;a_{2}\;e^{b_{2}\;t} \end{array}$$ (7)

The structure of the motion is comparable with the step response of a second order over-damped spring-mass-damper system, as the exponents of both terms are negative and less than 1 as per definition. However, the steady state gain is not equal for both terms but it differs by a factor of 10 for each exponential. This suggests that the settling dynamics is similar to a second order system but the steady state differs. However, in our case, once the data reach its steady state the fourth phase starts.

## Discussion

It is commonly agreed that the approaching to grasp motion follows a pre-defined timed plan, in terms of hand transportation and grip formation, which can be perturbed within limits [54, 55]. In this Section we contribute to this statement further.

It is worth observing that the first phase of our data has a bell-shaped form. This result is in line with many findings such as [51] and [56]. Specifically, authors in [51] also fitted a Gaussian model to their data as performed in this study, although the complexity of the model was higher probably due to the fact that the whole motion was involved. The study in [51] suggests that the reaching motion is an open-loop motion. Our findings, however, demonstrate that this open-loop profile terminates before the end of the motion. Marteniuk et al. [57] also observed a similar difference when subjects were asked to reach to a point or approach to grasp for lifting the object. The authors found that the hand decelerates longer for more complex tasks. Our findings also confirm the difference between reaching to a point and approaching to grasp.

Indeed, the open-loop reaching part of the motion has a defined duration after which the strategy of the approach motion is being defined. In this regards, the second phase is the moment when the final approach and grasping patterns are finalised. In agreement with Marteniuk et al. [57], this phase features a sharp deceleration whose shape is common across subjects, possibly because they all performed the same task. We found that the actual length of the phase is different for each subject and object being grasped, although this might be also caused by contingent factors during the experiment. Our results also found that most of the finger preshaping is performed in this phase. This finding is in line with what is observed by [49] and it complements our previous work [58] which describes how fingers are displacing for grasping. It is possible to observe that the precise approaching to grasp strategy is decided by the end of this phase.

The last phase is the third phase where the decided grasp posture and approach strategy are performed. Jeannerod [49], in a similar study involving approach to grasp for transporting, also observed that subjects undergo a low-velocity phase consistently at the same moment near the end of the motion. That study suggests that this phase is functional to prehension and is not a corrective action. Our results add to this statement. They suggest that the last phase is the only part of the motion which is reactive and where the finger joint speed variability is minimal. This suggests that the act of terminating the approach to grasp motion is a scripted mechanism.

The object-dependent phase of the approach motion, the fourth phase, possibly has a role in grasping while approaching. In this phase the object is gripped and lifted as the MCP joints are most active. Additionally, the fourth phase is the only part of an approaching motion which is performed in different ways. It is possible to speculate that the fourth phase might be involved in finalising the last details of a grasp affordance, such as completing the shape of the grip, in agreement with the results of [48]. In [48], the authors identified a common structure of the motion of the fingers when reaching to grasp which varies greatly in the last moments of the motion, when the object is about to be gripped. These results complement our findings on the common structure of the approaching motion and the lack of similarities on its fourth and last phase, demonstrating that a similar pattern can be observed in the motion of the fingers as well. A more through analysis of the finger motions is required to investigate how the grip is shaped in the fourth phase and relate it to approaching.

Additionally, it is worth to mention that some similarities can be observed between the velocity profile of the first two phases combined and the velocity profile of subjects reaching to grasp when their vision is impaired. Subjects with impaired vision have to perform anticipatory motion control to successfully manipulate an object, as some of its features, such as weight [59], are unpredictable. If vision is impaired unpredictably during reaching to grasp objects of varying shapes [60], or if subjects are blinded when reaching to grasp to pull an object [47], the speed profile differs from the that of subjects reaching to grasp with full vision. In the above-mentioned studies, the reaching motion was slower and the speed profile reached its peak earlier than when reaching with no impairments. In our study, the initial parts of subjects’ speed profiles also followed a similar structure, although no vision impairment was applied. A possible explanation could be that both in [47, 60] and in our study subjects were asked to perform some manipulation rather than simply reaching. In our case, complex finger control was required to complete the action, while in the other studies’ case fingers were used to determine haptic cues on the object used. This might influence the speed profile when reaching the object.

[61] and [62] observed that grasping kinematics and kinetics are independent activities that are planned in parallel when approaching to grasp. Our study agrees on the point that grasping and approaching seemed to be parallel operations. As the approaching is performed, the motion type changes from planned, to reactive, to object specific, as the grasp was about to be finalised. It can be speculated that the grasping finalisation gains an higher importance than approaching near the end of the motion, as the type of control changes. Our study is also in agreement with the findings of [61] that explicit (visual) knowledge of the object centre of mass allows subjects to modulate the approach to grasp. However, some objects, like the hammer, were familiar for the subjects, others, like the carton box, were of unpredictable weight and assumptions could be made on the characteristics of some objects (e.g. it is unlikely to grasp a full cup of coffee). It is therefore difficult to draw a conclusion on whether prior knowledge of the object or visual cues have a role in shaping the approaching to grasp overall motion structure. An additional study would be required to rule out the contribution of implicit or explicit knowledge when planning the approaching to grasp action.

The presented findings support the hypothesis that the approach to grasp motion follows mostly a planned strategy, although the last phase of the motion is a scripted and reactive component. Our results also support the hypothesis that the finger motion is synchronised with the hand motion, as most of the preshaping is performed in a specific phase.

## Conclusion

In this study, we defined two components of a grasp affordance: an initial approaching to grasp phase, and a second phase where the desired grasp pattern is implemented. The approach to grasp for hammering was studied, collecting data from 9 subjects who used very different objects, in different orientations, as hammers. The collected data was analysed and mathematical models, reliably describing the motion, were defined.

Our findings show that subjects share a common approaching to grasp pattern. Such pattern has a defined structure of three phases that can be reliably modelled mostly as a planned and intentional motion. The first two phases of the motion are part of a planned motion, while the third phase follows the dynamics of a spring-mass-damper system and is a reactive motion. The final action of grasping is performed in a fourth phase which does not have a common structure across subjects. We described the role of each of the first three phases in the discussion.

The proposed models of approaching can be used to provide a modular control policy for an approach motion controller for grasping to hammer. Since the overall model is structured in three individual models, it is possible to substitute one of the proposed model with an alternative one extrapolated from the data collected in this study. The control policy itself can be used to control a robotic end effector. An algorithm can be used to post-process the output so that it is suitable for a specific robotic control technique [63]. For example, the model’s profile could be used to shape an attractor landscape which would drive the hand towards the object’s centroid [64]. This will reduce the use of geometrical features of the object for the hand control. Additionally, the proposed approach model can be used as a starting point to derive other control policies for similar actions. For instance, it could be possible to derive a control policy for grasping to insert action, since inserting and hammering are similar. The new policy could be derived by adjusting the parameters of the proposed model with trial and errors using any reinforcement learning algorithm [65]. As future work, the proposed models and the learning algorithm will be implemented on a real robot as approaching policy.

The proposed approach is generalisable to different grippers, from traditional hard robotic hands to soft hands and even industrial end effectors, such as suction cups or soft manipulators [66]. Such flexibility is possible because the approaching models are using the centre of the end effector to control the hand, abstracting the specific details of the hand’s kinematics. However, soft underactuated hands [67, 68] are best suited to the proposed approach. The nature of those hands allows to passively shape and adapt the grasp posture while gripping the object [69], taking advantage of the physical constraints imposed by the environment [70]. The mechanical design of such hands and the elasticity of their tendons [71] protect the end effector from breaking in case of involuntary collision with a surface or the object. Therefore, soft hands can compensate for an imprecise approaching strategy as they adapt their shape or push the object in-hand when the grasp is executed [72]. Additionally, pairing a soft hand with our approach could replace the need for a model for phase 4, the object-dependent part of an approaching to grasp motion. It is possible to speculate that humans adapt their hand posture at grasping time to take advantage of the environment [73], rather than only relying on a pre-encoded set of grasp affordances. Soft hands offer a similar capability by design. Hence, if the environment does play a role in adjusting the grasp pattern, a soft hand could provide a model-free grasp finalisation to complement our model. Additionally, such pairing could drastically reduce the use of ad-hoc precomputed grasp affordances or approaching profiles [74]. Also, the computational complexity of object perception algorithms [75], which often rely on expensive and power intensive ad-hoc processors (i.e. Graphical Processing Units), would be simplified since only an approximate shape, position and orientation would be required to approach and grasp an object. Hence, less prior information on the characteristics and outlook of an object would be required, allowing the robot to better operate in unstructured environments, such as houses or public venues, where the used objects are too many to be all modelled.

It is worth mentioning that this paper analyses only the distance between hand and object, but the relation between hand and object orientations is not discussed. This choice is justified by the fact that understanding the fundamental principles of hand trajectory generation in human approaching is the first step required to translate those principles in approaching to grasp for robotics. This would give the opportunity to reduce the need of prior knowledge of the proprieties of the manipulated object, which is an unsolved challenge in robotic manipulation. However, the hand-object orientation relation should also play a role in defining the structure of approaching to grasp as it is expected to influence the second part of a grasp affordance as well. Such data is currently being analysed and will be presented as future work in a dedicated study. Preliminary insights on this subject, are presented in [63]. It was found that the actual length of the phases is different from subject to subject. This factor is more significant for studies of human behaviour than for robotics as the duration of an execution is often configurable and influenced by technical details. Therefore, this can be explored in future research using the collected dataset. Additionally, the current study analyses direct approaching to grasp patterns. There might be alterations to the patterns in presence of obstacles, constraints or other impairments such as lack of vision or tactile sensation. Also, a different action, or the same action involving flexible or deformable objects, might have a different structure that might need to be modelled differently. Those factors would require a separate study.

Finally, it shall be underlined that the models proposed in this paper are the most reliable representation of the data. This means that it is expected that the selected models have the same good performance for any input as they are meant to describe the phenomena. A robotic implementation might not require the same level of precision, as the objective is to replicate the functionality of the human motion. As such, simpler polynomial versions of the proposed models can be adopted as well as compared against the high precision ones proposed in this paper. Additionally, a criterion to switch between models of different phases is needed for the motion to be smooth. In this sense, it is possible to interpolate the last point of one model and the first one of the subsequent model. The validity of such criterion has to be validated in a robotic implementation, and the approach was tested in simulation in [63]. These analyses will be part of future works.

The proposed models and characterisation of grasp affordance, in terms of hand speed and acceleration when approaching an object for using it, underline the importance of the actual action being performed over the features of the object being handled. Since the approaching pattern is general and object independent, only a different action would require a different grasp affordance pattern. As such, the action to be performed, rather than the manipulated object, should be the discriminant in deciding the specific grasp posture and approaching motion to be employed among many.
